# Non-pharmacological therapies for treating chronic pelvic pain in women: A review

**DOI:** 10.1097/MD.0000000000031932

**Published:** 2022-12-09

**Authors:** Xinlu Wang, Ning Ding, Yuanjie Sun, Yu Chen, Hangyu Shi, Lili Zhu, Shuai Gao, Zhishun Liu

**Affiliations:** a Department of Acupuncture, Guang’anmen Hospital, China Academy of Chinese Medical Sciences, Beijing, China; b New Zealand College of Chinese Medicine, Greenlane, Aukland, New Zealand; c Beijing University of Chinese Medicine, Beijing, China.

**Keywords:** acupuncture, chronic pelvic pain, non-pharmacological therapy, psychotherapy, therapeutic intervention

## Abstract

Chronic pelvic pain (CPP) is an intricate condition with multiple etiologies that lead to indefinite pain mechanisms. Physicians and researchers are challenged in its treatment, and the combined therapy of pharmacologic and non-pharmacologic treatment has been recognized as a multidisciplinary approach cited by guidelines and adopted in clinical practice. As an alternative therapy for CPP, non-pharmacologic therapies benefit patients and deserve further study. This study reviews the literature published from January 1991 to April 2022 on non-pharmacologic therapies for CPP in adult women. Based on a survey, this review found that the most commonly used non-pharmacological therapies for CPP include pelvic floor physical therapy, psychotherapy, acupuncture, neuromodulation, and dietary therapy. By evaluating the efficacy and safety of each therapy, this study concluded that non-pharmacological therapies should be included in the initial treatment plan because of their high degree of safety and low rate of side effects. To fill the lack of data on non-pharmacologic therapies for CPP, this study provides evidence that may guide treatment and pain management.

## 1. Introduction

Chronic pelvic pain (CPP) in women, defined by the American College of Obstetricians and Gynecologists, is pain derived from the pelvis, lasting for more than 6 months, which results in negative psychology, dysfunctional sexuality and behavior, and lower urinary tract symptoms.^[1]^ In severe cases, CPP, identified as a symptom rather than a disease,^[2]^ involves non-cyclical pain accompanied by dyspareunia and dysmenorrhea.^[3]^CPP is estimated to affect 5.7% to 26.6% of women in the world.^[1,4]^ In the US, CPP causes almost 15% female off-work and a 45% decrease in working productivity.^[5]^ CPP in the US accounts for 10% of gynecologic clinic visits and 40% of laparoscopies,^[5,6]^ which cost $880 million annually.^[5]^ If combined with the cost of treating individual conditions related to CPP, the cost is estimated to reach $289 billion.^[3]^

However, the etiology of CPP is poorly understood, because not only 1 factor or disorder is attributed to pain.^[6,7]^ According to the American College of Obstetricians and Gynecologists guidelines, CPP may be caused by multiple factors that interact with each other, including visceral etiologies, neuromusculoskeletal disease, and psychosocial status.^[1]^ CPP is related to both gynecological and non-gynecological disorders such as endometriosis, intra-abdominal adhesions, irritable bowel syndrome (IBS), and interstitial cystitis/painful bladder syndrome.^[1,7,8]^

Considering the complicated causes and limited options for CPP treatment, physicians must have to focus on reducing symptoms in advance of curing the patient completely.^[7]^ Pharmacologic and surgical therapies are the most popular options for treating CPP, but they are not cost effective and have always been reported to have side effects such as bloating, drowsiness, and constipation.^[7,9,10]^ Current guidelines advise the use of a multidisciplinary team (MDT) approach to improve therapeutic effect.^[1,3,7]^ Previous studies have noted that the early use of non-pharmacologic therapy could complement CPP treatment in terms of alleviating pain and avoiding drug addiction.^[1,3]^

Non-pharmacologic therapies, with low risk of side effects, are beneficial to patients suffering from drug intolerance, multiple comorbidities, and refractory pain.^[7]^ Meanwhile, it alleviates psychological symptoms, such as depression and anxiety.^[11–14]^ A prospective study showed that more than 50% of patients with CPP are likely to pursue non-pharmacologic therapies.^[15]^ Therefore, this study reviews non-pharmacologic therapies for CPP in women, including pelvic floor physical therapy, psychotherapy, acupuncture, neuromodulation, and dietary therapy (Table 1).

**Table 1:** Key outcomes of nonpharmacologic therapies for CPP.

Yr intervention	Study design N at enrollment (N at follow-up)	Treatment period follow-up period	Results
2012 Myofascial physical therapy vs global therapeutic massage	A randomized multi-center clinical trial Myofascial physical therapy:42(40) global therapeutic massage:39(38)	12-wk 3 mo	59% response* in myofascial physical therapy group;26% response in global therapeutic massage group (*P* = .0012)
2020 Group-based multimodal physical therapy vs primary-care physical therapy	A randomized controlled trial Group-based multimodal physical therapy:32(26) primary-care physical therapy:30(25)	16-treat days 9-mo	Difference change of mean pain intensity score in groups: −1.2 (*P* = .027)
2016 Psychotherapy with somatosensory stimulation (different techniques of acupuncture point stimulation) vs waitlist control	A randomized controlled trial Psychotherapy with somatosensory stimulation (different techniques of acupuncture point stimulation):35(30) waitlist control:32(26)	3-mo 24-mo	Difference change in 2 group: Maximal global pain (−2.1, *P* = .002); Ave- rage global pain (−2.5, *P* < .001); Pelvic pain (−1.4, *P* = .036); Dys- phasia: (−3.5, *P* = .003)
2020 Ashi acupuncture vs local anesthetic trigger point injection	A randomized clinical trial Ashi acupuncture:16(16) local anesthetic trigger point injection:19(19)	G1:10-wk G2:14-wk 6-mo	Same reducing clinical pain in Ashi acu-puncture and local anesthetic injection
2021 Manual acupuncture plus usual care vs usual care	A randomized con-trolled feasibility study Manual acupuncture plus usual care:16(12) usual care:15(7)	8-wk 7-mo	Decrease Pain score 1.9 point in Manual acupuncture; no decrease in usual care
2014 Percutaneous tibial nerve stimulation group vs control group	A randomized trial Percutaneous tibial nerve stimulation group:16(16) control group:17(17)	12-wk 6-mo	Decrease pain severity and improve quality of life in PTNS
2014 Transcutaneous electrical nerve stimulation vs placebo	A prospective, experimental study Transcutaneous electrical nerve stimulation:92(92) placebo:30(30)	2-wk 4-wk	Improve pain scores in TENS group

response*: the rate of responders who improved or markedly improved in overall symptoms compared to baseline on a 7-point global response assessment.

### 1.1. Pelvic floor physical therapy (PFPT)

Pelvic floor physical therapy (PFPT) focuses on mitigating tenderness of the muscles in the pelvic floor, back waist, abdominal wall and hips.^[1,7,10]^ Dysfunction of the pelvic floor musculoskeletal system is believed to be the primary cause of CPP, compared to CPP, which is secondary to pathology elsewhere in the human body.^[2]^ As per patients’ symptoms, physical therapists adopt various interventions, such as pelvic floor muscle stretch, myofascial release, manual therapy, biofeedback and electrotherapy.^[16–18]^

The rationale behind PFPT is to invigorate blood flow in the pelvic floor area, ease and stretch contracted muscles, release rigid articulations, and increase elasticity of the soft tissues. As a result, pelvic floor muscle function is expected to be restored, with pain reduced as much as possible.^[13,19,20]^ Obviously, PFPT suits the patients with pelvic floor muscle dysfunction, and is especially effective in treating recurrent tenderness in contracted pelvic floor muscles.^[7,17,21]^

Although clinical experience indicates that PFPT is effective, evidence-based reports are rare in the literatures.^[7]^ A pilot randomized controlled trial in women with pelvic floor myalgia suggested that PFPT reduces pain more significantly than levator ani muscle trigger-point injections.^[22]^ Another clinical trial showed that myofascial physical therapy benefits women with pelvic floor tenderness.^[23]^ A randomized controlled trial concluded that group-based multimodal physical therapy based on the biopsychosocial model eases pelvic pain more significantly than primary-care physical therapy.^[21]^

Considering the limited therapeutic effect of PFPT, patients with CPP should be advised in the initial treatment to be aware of the potential etiology, illness duration, possible delayed discomfort effect and time commitment.^[7]^

### 1.2. Psychotherapy

Psychological factors play an important role in triggering CPP, especially in women^[11,24]^ who experience negative emotions such as depression, anxiety, catastrophizing and post-traumatic stress disorder.^[10,24]^ Psychotherapy is an effective, safe, and noninvasive treatment for women with CPP that can change the cognition, mood, and behavior of patients.^[7]^ Cognitive behavioral therapy (CBT), a psychotherapy for CPP in women, is effective and widely used in treating chronic pain conditions (SOGC recommendation, grade 1 evidence).^[25]^

As a goal-directed psychotherapy,^[10]^ CBT guides patients to acknowledge pain and alter thoughts and behaviors to deal with pain.^[1,10,26]^ To restructure cognitions and modify patients’ behavior or lifestyle, CBT leverages techniques such as time-based pacing, sleep hygiene, muscular relaxation, training in meditation, stress managements, and appropriate communication with physicians, friends and family.^[10,14,25]^

Although the mechanisms by which CBT benefits patients with chronic pain are still uncertain, some studies have hypothesized that CBT changes the gray matter responsible for pain management in the brain.^[27,28]^ CBT decreases the severity and frequency of pain symptoms by reducing stress and by altering catastrophic cognition. A 1-year follow-up study indicated that CBT combined with gynecologic care could reduce psychological symptoms, pain, and related dysfunction in chronic pelvic viscera and muscle^[29,30]^ and the effect could last for 9 months.^[30]^ Similarly, a randomized controlled trial reported that psychotherapy with somatosensory stimulation reduced pelvic pain, relieved constipation, and improved the quality of life of patients with endometriosis-associated pelvic pain.^[31]^

Besides CBT, another psychotherapy of mindfulness could also promote tolerance of pain by reducing depression and stress in CPP patients.^[7]^ Mindfulness focuses on improving sensations of pain and related responses, instead of reducing pain itself.^[12]^ Moreover, mindfulness benefits from chronic pain, fibromyalgia, and IBS.^[12,32–35]^ Another pilot study showed that 8-week mindfulness produces significant improvements in daily pain scores from baseline and thus improves the quality in CPP patients.^[36]^ Therefore, psychotherapy is effective in treating CPP complicated by psychological disorders^[10]^

### 1.3. Acupuncture

Acupuncture, identified as an independent treatment modality, has been widely accepted for managing pain, including muscular and myofascial pain, lumbar and pelvic pain in primigravida, and chronic prostatitis/CPP symptom.^[27,37–39]^ In addition, acupuncture has been approved by the FDA for the treatment of tumor pain.^[25]^ A systematic review of 6 randomized trials showed strong evidence of the effectiveness of acupuncture in reducing pain and decreasing opioid use in patients with chronic musculoskeletal pain.^[1]^

The mechanism of acupuncture analgesia is not clear, but studies have shown that neuroendocrine, immunological, and cardiovascular reactions can be triggered by acupuncture that penetrates the peripheral system, producing mechanical signals and generating cascaded effect.^[40,41]^ Acupuncture can balance homeostasis by stimulating sympathetic neurons and somatic afferent fibers of neurons.^[41]^ Acupuncture can also form a cutaneous microcurrent to promote tissue growth.^[42]^ Unlike morphine, which develops drug tolerance in patients with chronic pain, multiple sessions of acupuncture do not inhibit the reaction to needling. Acupuncture may promote endogenous opioid release and strengthen gate control in the pain pathways.^[25]^

A 3-arm randomized controlled pilot study concluded that meridian balance method acupuncture plus electroacupuncture treatment could relieve pain and improve physical and emotional functions in women with CPP.^[43]^ Another randomized sham-controlled trial also showed that Japanese-style acupuncture is safe, effective, and non-addictive for treating endometriosis-related pelvic pain in women.^[44]^ Studies also indicate that Ashi acupuncture is as effective as local anesthetic injections in easing pain in women with CPP,^[41]^ and manual acupuncture plus usual care is more acceptable, safe, and effective than usual care alone.^[45]^ In addition, a randomized controlled trial concluded that neurogenic acupoint cupping, a traditional antient treatment, could significantly reduce inflammation, pelvic pain, and improve quality of life in women with CPP.^[46]^ These studies suggest that acupuncture is an effective and safe non-pharmacological therapy for CPP in women.

### 1.4. Neuromodulation

Various peripheral and central neuromodulation therapies have been widely studied for chronic pain control.^[7,47]^ Peripheral neuromodulation includes percutaneous tibial nerve stimulation (PTNS), transcutaneous electrical nerve stimulation (TENS), sacral neuromodulation, and pudendal neuromodulation.^[47]^ Peripheral neuromodulation stimulates peripheral Aβ-fibers and suppresses Aδ-fiber nociceptors, thus reduces pain.^[48]^ A study believes that Neuromodulation can alter nerve conduction and attenuate pain sensation by stimulating microcurrent.^[7]^ Central neuromodulation, such as transcranial direct current stimulation (tDCS), alleviates pain by producing microcurrents that promote neuronal excitability and activate descending inhibition systems.^[49]^

PTNS was performed by inserting a 34-gauge needle approximately 3 to 4 cm above the medial malleolus and 1 cm posterior to the tibia, along the running direction of the tibial nerve, placing the adhesive electrode on the medial heel, and then adjusting the stimulation amplitude, typically 30 min per session for consecutive 12 weeks.^[47,50,51]^ A prospective study showed that PTNS could decrease pain and improve quality of life in women with CPP.^[51]^ A randomized trial showed that PTNS is an effective therapy, with minimal invasion and a long-term efficacy of 6 months.^[50]^

TENS suppresses pain by delivering a low voltage and low electric current from removable skin electrodes. Incorporated with pelvic floor physical therapy, TENS has been identified as the most frequently used noninvasive therapy.^[7,47]^ A prospective study showed that TENS is a safe, economical, and useful intervention for reducing pain.^[18]^ A feasibility study proved that TENS is effective for long-term stability.^[52]^

Sacral and pudendal neuromodulation plants a programmable stimulator subcutaneously to deliver stimulation to sacral or pudendal nerves, decreasing somatic and sympathetic signals.^[47,53]^ A case report showed that sacral neuromodulation decreases the severity and frequency of CPP over a period of 19 months after implantation.^[54]^ A single-cohort pilot study reported that pudendal neuromodulation could relieve pain to different extents.^[55]^ Central neuromodulation of tDCs is usually applied in treating psychiatric conditions,^[56]^ but a double-blind sham-controlled crossover study showed that it benefits women with CPP by reducing pain and improving quality of life.^[57]^

Despite the variety of neuromodulations that relieve the symptoms of CPP, more high-quality evidence is needed to clarify its function and rationale.

### 1.5. Dietary therapy

As a self-care management regimen, dietary therapy can potentially benefit patients with CPP.^[3]^ Patients are encouraged to consume gluten-free and anti-inflammatory foods, with low sugar and salt,^[7]^ meanwhile avoiding acidic or pungent foods.^[38]^ However, limited evidence in the literature supports its efficacy in the treatment of CPP in women.^[7]^ A cohort study showed that a gluten-free diet alleviates CPP in 75% of subjects over a follow-up period of 12 months.^[58]^ A systematic review found that persistent diet-control and clear diet intervention by experienced dieticians is critical in CPP treatment.^[59]^ Unlike other non-pharmacological treatments, dietary therapy is expected to be performed by patients on their own.

## 2. Conclusion

In the initial treatment of CPP, it is crucial to clarify all possible etiologies because only a single etiology-treatment hardly relieves pain, which could disappoint patients and physicians. Regardless of the therapies used, pharmacologic, non-pharmacologic, or surgical therapy, no single definite approach can cure CPP; therefore, a multidisciplinary approach should be adopted.

Non-pharmacologic therapy should be planned and applied initially and at each stage of CPP treatment. It is beneficial to first consider psychotherapy, as it is a representative non-pharmacologic intervention that modulates the thoughts and behaviors of patients, especially in women with negative emotions. By virtue of the specialty and safety of non-pharmacologic therapy, this study suggests introducing non-pharmacologic therapy as early as possible in treatment. Non-pharmacologic therapy contributes to a multidisciplinary approach with multiple choices of pain management. While evidence of non-pharmacologic therapy is increasing in the literature, more evidence-based studies with quantitative analysis are needed.

## Author contributions

All the authors listed have made an intellectual and substantial contribution to the work and supported it for publication.

**Data curation:** Xinlu Wang, Yuanjie Sun.

**Formal analysis:** Zhishun Liu.

**Investigation:** Xinlu Wang, Hangyu Shi.

**Resources:** Xinlu Wang, Lili Zhu.

**Supervision:** Shuai Gao.

**Writing – original draft:** Xinlu Wang, Zhishun Liu.

**Writing – review & editing:** Xinlu Wang, Yu Chen, Zhishun Liu.
